# Supplementary material for: How does a preference-based generic health-related quality of life measure perform in patients with a rare disease? Measurement properties of the EQ-5D-Y proxy version among underage patients with neurofibromatosis type 1
Source: Orphanet J Rare Dis. 2025 Feb 13;20:71. doi: 10.1186/s13023-025-03590-x (PMC11827137; doi:10.1186/s13023-025-03590-x)
Supplement: Supplementary file 1 — Supplementary Material 1 [file 13023_2025_3590_MOESM1_ESM.docx]

How does a preference-based generic health-related quality of life measure perform in patients with a rare disease? Measurement properties of the EQ-5D-Y proxy version among underage patients with neurofibromatosis type 1

Wanxian Liang^1,2^, Shihuan Cao^1,2^, Yusi Suo^1,2^, Lining Zhang^1,2^, Lujia Yang^1,2^, Hanfei Wang^1,2^, Han Wang^1,2^, Xuejing Jin^1,2*^

^1^Center for Evidence-Based Chinese Medicine, School of Traditional Chinese Medicine, Beijing University of Chinese Medicine, Beijing, China

^2^International Institute of evidence-based traditional Chinese medicine, Beijing University of Chinese Medicine, Beijing, China

*Corresponding author. E-mail(s): jinxuejing2018@163.com;
Contributing authors: wxliang1995@outlook.com; shcbucm@163.com; suoyusi716@163.com; zhang.ln@outlook.com; yanglujia45@163.com; wanghanfei2020@163.com; Chen_cuncun@163.com

Abstract

**Background** EQ-5D-Y is a pediatric preference-based health-related quality of life (HRQL) measure that is recommended in health economic evaluation according to China’s guidelines. However, there is limited evidence regarding how the EQ-5D-Y perform in patients with rare diseases in the country. Neurofibromatosis type 1 (NF1) is a rare disease that affects the growth and development of underage patients. This study aimed to examine the performance of EQ-5D-Y proxy version among underage NF1 patients in China.

**Methods** Data from a nationwide cross-sectional survey from Nov 2022 to Jan 2023 was used. A total of 154 caregivers for underage NF1 patients who completed the EQ-5D-Y proxy version, PedsQL 4.0 Generic Core Scales (PedsQL GCS) proxy version, and Zarit Burden Interview (ZBI-22) were included. The performance of the EQ-5D-Y was assessed by response pattern (ceiling and floor effects), convergent validity against the PedsQL GCS, known-groups validity, and Shannon (H’) and Shannon evenness (J’) indices.

**Results** Data from 154 caregivers were analyzed. The mean age of caregivers was 36.66 (6.18) years, and 80.27% of them were mothers of NF1 patients. The mean age of NF1 patients was 8.38 (3.34) years, with 51.30% being females. The ceiling effect of EQ-5D-Y was 30.52%, and floor effect was 0%. Moderate to strong correlations were found between EQ-5D-Y and PedsQL GCS dimensions that share similar constructs (rho -0.42 to -0.60, all *p* values < 0.001). The hypotheses of known-groups defined by different PedsQL GCS and ZBI scores were validated. The EQ-5D-Y exhibited the strongest informativity and discriminatory power of the “feeling worried, sad or unhappy” dimension and weakest for the “mobility” dimension (H’_(mobility)_=0.60, J’_(mobility)_=0.38; H’_(feeling worried, sad or unhappy)_=0.60, J’_(feeling worried, sad or unhappy)_=0.38).

**Conclusions** EQ-5D-Y is acceptable for measuring HRQL of underage NF1 patients in China. More evidence for using EQ-5D-Y in rare diseases is awaited.

Keywords: EQ-5D-Y; Measurement properties; Neurofibromatosis type 1; Health-related quality of life; Rare diseases

# Introduction

There are more than 7,000 rare diseases around the world[1]. Most rare diseases are genetically related, with approximately 70% having childhood-onset [2]. This often results in lifelong physical and psychological distress for both patients and their families [2]. Rare diseases can be life-threatening. The mortality rate for affected individuals under five is around 30% [3]. Neurofibromatosis type 1 (NF1) is a rare disease occurring in every 3000 newborns globally [4]. The clinical manifestations of NF1 can vary widely and may worsen over time. Common symptoms include café-au-lait spots and tumors along nerves affecting multi-systems in the body. While mild cases may only exhibit skin symptoms, severe cases can lead to life-threatening conditions. The dermatological manifestations can affect the psychological health of NF1 patients, while other internal manifestations, such as neurofibromas, can affect their physical health [5]. Rare diseases in China are managed through an officially defined catalog of rare diseases. NF1 is one of the diseases included in China’s Second Catalog of Rare Diseases [6].

In recent years, increasingly number of orphan drugs have been developed [7, 8], bringing hope for patients with rare diseases while usually with high prices. Including expensive orphan drugs in the reimbursement catalog allows more patients with rare diseases to access these medicines, ensuring they truly benefit from them. In many countries such as the UK, Canada, Japan, Australia, Sweden, and China, economic evidence is required for drug reimbursement decision [9-12]. Cost-utility analysis, which typically employs preference-based health-related quality of life (HRQL) measures, is the most recommended analytical technique for economic evaluation in all those countries mentioned above [13]. However, numbers of pediatric-specific preference-based HRQL measures are limited. Common used pediatric-specific preference-based HRQL measures are EQ-5D-Y, Child Health Utility 9D (CHU9D), and Health Utilities Index (HUI) [14, 15]. According to the pharmacoeconomic evaluation guidelines of the UK, Australia, and China, suitable pediatric-specific HRQL should be used for children in pharmacoeconomic evaluations. China Guidelines for Pharmacoeconomic Evaluations (2020 Edition) specifically pointed out that the EQ-5D-Y is recommended for children [16]. The EQ-5D-Y is the youth version of the EuroQol five-dimensional (EQ-5D) descriptive system, designed for children and adolescents aged 4 to 18 [17]. It features 3 response levels for each dimension to assess physical and mental health. The EQ-5D-Y retains the content and structure of the standard EQ-5D, facilitating a seamless evaluation of health status from childhood into adulthood [18]. EQ-5D-Y is increasingly popularly used to measure HRQL in various pediatric populations [19-24].

Even though EQ-5D-Y has been used among patients with NF1 and other rare diseases [17, 25-28], it hasn’t been validated in China’s NF1 populations. Given China’s large population, the number of NF1 patients is expected to be significant in the country. Assessing measurement properties of the EQ-5D-Y in Chinese patients with NF1 can support medical decision making in this population. The present study aimed to investigate the measurement properties of EQ-5D-Y among underage patients with NF1 in China. The findings will provide insights into the performance of EQ-5D-Y in rare disease populations.[7][29][8] [30][6, 31, 4][5][19, 20, 32-34][25-28]

# Methods

## Data collection

The study used data from a cross-sectional survey conducted from Nov 2022 to Jan 2023. In this survey, primary caregivers for underage NF1 patients were recruited through a nationwide patient group of the NF1 population, Neurofibromatosis Shenzhen Care Center (NSCC). Volunteers from NSCC helped to organize the participants and make preparations for the survey.

As per the ethics committee’s recommendations, all questionnaires in this study should be completed by caregivers of NF1 patients acting as proxies for NF1 patients who were under 18 years old, even though there are varying opinions regarding the consistency between proxy-reported and self-reported HRQL [29-32]. Early studies showed that the proxy and self-reported versions of EQ-5D-Y and PedsQL GCS generally demonstrate moderate to good agreement [33-39]. Caregivers were asked to fill out a questionnaire using an online survey platform, where researchers could monitor the entire process of answering the questions. Information on medical treatment (e.g., annual outpatient visit times and hospitalization times) in the year 2021 was collected. The EQ-5D-Y proxy version, PedsQL 4.0 Generic Core Scales (PedsQL GCS) proxy version and Zarit Burden Interview (ZBI-22) were used. The EQ-5D-Y was applicable for caregivers of those patients aged from 4 to 18, and PedsQL GCS was applicable for caregivers of those patients aged from 2 to 18. ZBI-22 was applicable for all the caregivers included. A total of 223 caregivers of NF1 patients were recruited in the cross-sectional survey, only those who completed both the EQ-5D-Y and PedsQL GCS were included in the present study (n=154).

Ethics approval was obtained from the Ethics Committee of Beijing University of Chinese Medicine (2022BZYLL1005). The study was carried out according to the Declaration of Helsinki. Written informed consent was obtained before the survey began.

## Measures

In this study, the EQ-5D-Y proxy version and PedsQL GCS proxy version were used to assess HRQL of underage NF1 patients, and ZBI-22 was used to assess caregiver burden.

### EQ-5D-Y

The EQ-5D-Y is a youth version of the EQ-5D descriptive system, designed for children and adolescents aged 4 to 18, and it has a value set for China [17]. Caregivers filled out EQ-5D-Y as proxies only for patients aged between 4 and 18 years old in this study. [46]EQ-5D-Y collects information on one’s health status from three response levels (no problems, some problems, and a lot of problems) of five dimensions: “mobility”, “looking after him/herself”, “doing usual activities”, “having pain or discomfort”, “feeling worried, sad or unhappy”[40]. A visual analog scale (VAS), ranging from 0 to 100, is presented to the respondents after they fill out their health status of five dimensions to get the subjective feeling of their total health. Previous studies demonstrated good validity and reliability of EQ-5D-Y in underage populations [22, 27, 28, 41-43].

In the present study, caregivers’ responses to the EQ-5D-Y were converted from the five questions to preference-based EQ-5D utility scores using a value set for EQ-5D-Y in China [17].

### PedsQL 4.0 Generic Core Scales

PedsQL GCS is a 23-item HRQL measure for children aged 2 to 18, using 5-point response Likert scales to assess the health status [44]. PedsQL GCS showed good reliability and validity in different populations including NF1 patients and was recommended to be used in neurofibromatosis trials [45-49]. In the present study, caregivers filled out PedsQL GCS proxy version if patients aged between 2 and 18 years old. There are 4 scales in PedsQL GCS assessing HRQL in different domains: 1) physical functioning (8 items), 2) social functioning (5 items), 3) emotional functioning (5 items), and 4) school functioning (5 items). Total score and scale scores of PedsQL GCS were calculated as the average of all the items in the whole PedsQL GCS and each scale and then reversely and linearly transformed into scores ranging from 0 to 100, using the scoring system of the PedsQL questionnaires. A higher PedsQL total score or scale score means a better health status.

### Zarit Burden Interview

Zarit Burden Interview (ZBI-22) is a 22-item questionnaire used to measure a caregiver’s burden. ZBI-22 has been used to assess the caregiver burden of different population including NF1 patients and proved to be valid and reliable [50-55]. ZBI-22 uses a 5-level (0-4 points for each item) severity scale to evaluate how the caregiver feels when they provide care for the one being cared for. The total score of ZBI-22 ranges from 0 to 88. The higher the ZBI-22 total score is, the heavier distress a caregiver is experiencing. All caregivers included in the cross-sectional survey filled out ZBI-22.

## Data analysis

In the present study, measurement properties of the EQ-5D-Y were assessed by response patterns, construct validity, and informativity and discriminatory power.

Response patterns were demonstrated by ceiling and floor effects, and distribution across response levels of each dimension.

Construct validity included convergent validity and discriminant validity. Convergent validity was examined by Spearman’s correlation coefficient (rho) against the PedsQL GCS. Significant correlations were expected between similar constructs of EQ-5D-Y and PedsQL GCS. At the domain level, similar constructs included EQ-5D-Y “mobility”, “looking after him/herself”, “doing usual activities”, and “having pain or discomfort”, in correspondence to PedsQL physical functioning; “feeling worried, sad or unhappy” in correspondence to PedsQL emotional functioning; “doing usual activities” in correspondence to PedsQL school functioning and social functioning; “doing usual activities”, “having pain or discomfort”, and “feeling worried, sad or unhappy” in correspondence to PedsQL psychosocial health score [56]. Spearman’s correlation was identified as strong correlation (rho >0.7), moderate correlation (0.4 ≤ rho ≤ 0.7), and weak correlation (rho < 0.4) [57].

Discriminant validity was examined by known-groups validity. When analyzing known-groups validity, we analyzed known-groups as follows: 1) gender; 2) outpatient visit times (<5 times/year vs. ≥5 times/year); 3) median of ZBI-22 total score; 4) median of PedsQL GCS dimensions and pain scores. These groups are known to differ in health status, and it was anticipated that the EQ-5D-Y utility scores and VAS scores could distinguish children with different health statuses.

Informativity and discriminatory power were examined by Shannon (H’) and Shannon evenness (J’) indices.

In the study, data of EQ-5D-Y, PedsQL GCS, and ZBI-22 need to be complete in order to assess the measurement properties of EQ-5D-Y. Therefore, listwise deletion would be conducted if there was any missing data in EQ-5D-Y, PedsQL GCS, or ZBI-22. We conducted statistical analyses using Stata 17.0 SE. We considered that significant differences exist when *p* value was ≤ 0.05. Effect size is interpreted as small (Cohen’s *d* = 0.2), medium (Cohen’s *d* = 0.5), and large (Cohen’s *d* = 0.8).

# Results

A total of 223 primary caregivers for NF1 patients under 18 years old were recruited in the cross-sectional study. Because the EQ-5D-Y was applied for caregivers of NF1 patients no less than 4 years old, a total of 154 caregivers responded to all the three HRQL instruments (EQ-5D-Y, PedsQL GCS, and ZBI-22) and were included in the present study (Table 1). Since the data was collected by a one-on-one online survey to guarantee the quality of the research, participants in the study were required to answer all questions of EQ-5D-Y, PedsQL GCS, and ZBI-22 that were applicable. Therefore, there was no missing response of EQ-5D-Y, PedsQL GCS, or ZBI-22 in the present study.

Table 1. Characteristics of participants

|  | Variable | NF1 patients aged 0 to 18 | NF1 patients aged 4 to 18 |
| --- | --- | --- | --- |
|  |  | *n*=223 | *n*=154 |
| **Patient** | **Age, mean (SD), years** | 6.26 (4.25) | 8.38 (3.34) |
|  | **Disease duration, mean (SD)** | 2.81 (2.90) | 3.58 (3.14) |
|  | **Gender, (%)** |  |  |
|  | Female | 121 (54.26%) | 79 (51.30%) |
|  | Male | 102 (45.74%) | 75 (48.70%) |
|  | **Familial NF1, (%)** |  |  |
|  | No family member diagnosed with NF1 | 190 (85.20%) | 127 (82.47%) |
|  | More than 1 family member diagnosed with NF1 | 33 (14.80%) | 27 (17.53%) |
|  | **Annual outpatient visit times, (%)** |  |  |
|  | <5 outpatient visit times in 2021 | 184 (82.51%) | 126 (81.82%) |
|  | >=5 outpatient visit times in 2021 | 39 (17.49%) | 28 (18.18%) |
|  | **Annual hospitalization times, (%)** |  |  |
|  | No hospitalisation in 2021 | 175 (78.48%) | 113(73.38%) |
|  | More than 1 hospitalisation in 2021 | 48 (21.52%) | 41 (26.62%) |
| **Proxy^*^** | **Age, mean (SD), years** | 36.66 (6.18) | 38.23 (6.02) |
|  | **Character of caregiver, *n* (%)** |  |  |
|  | Mother | 179 (80.27%) | 121 (78.57%) |
|  | Father | 40 (17.94%) | 29 (18.83%) |
|  | Others^#^ | 4 (1.79%) | 4 (2.60%) |
|  | **Caregiver diagnosed with NF1, *n* (%)** |  |  |
|  | Yes | 18 (8.07%) | 15 (9.74%) |
|  | No | 205 (91.93%) | 139 (90.26) |
|  | **Marital status, *n* (%)** |  |  |
|  | Married | 203 (91.03%) | 136 (88.31%) |
|  | Others^##^ | 20 (8.97%) | 18 (11.69%) |
|  | **Employment, *n* (%)** |  |  |
|  | Employed full-time | 131 (58.74%) | 80 (51.95%) |
|  | Not employed | 32 (14.35%) | 26 (16.88%) |
|  | Employed part-time | 26 (11.66%) | 22 (14.29%) |
|  | Others^###^ | 34 (15.25%) | 26 (16.88%) |

Note: *NF1*, neurofibromatosis type 1.

^*^ A proxy in this study is the primary caregiver of a NF1 patient.

## Response Pattern

At the health state level, 30.52% of the respondents reported no problems in all five dimensions of EQ-5D-Y (“11111” state), which meant the overall ceiling effect for EQ-5D-Y was 30.52%. None of the respondents reported the most severe state in all five dimensions of EQ-5D-Y (state “33333”), which meant there was no floor effect for EQ-5D-Y (Table 2). At the dimension level, the proportion of the respondents who reported having problems was 11.69% for “mobility (waking about)”, 17.53% for “looking after himself/herself” and 27.92% for “doing usual activities”, 52.60% for “having pain or discomfort”, and 42.86% for “feeling worried, sad and unhappy”. The mean (SD) of EQ-5D-Y utility scores was 0.880 (0.133), and the mean (SD) of VAS scores was 75.38 (20.67).

Table 2. Response pattern of EQ-5D-Y, PedsQL GCS and ZBI-22 (n=154)

| Variable | mean (SD) | median (min, max) |
| --- | --- | --- |
| **HRQL** |  |  |
| **EQ-5D-Y** |  |  |
| EQ-5D Utility | 0.880 (0.133) | 0.910 (0.180, 1) |
| EQ-5D VAS | 75.38 (20.67) | 80.00 (0, 100) |
| **Proportion of reported problems in dimensions, %** |  |  |
| Mobility | 11.69 | |
| Looking after him/herself | 17.53 | |
| Doing usual activities | 27.92 | |
| Having pain or discomfort | 52.60 | |
| Feeling worried, sad or unhappy | 42.86 | |
| Proportion of state “11111” | 30.52 | |
| Proportion of very mild health state (response level of “6”, mild problems was reported in only one dimension, such as state “11112” or “21111”) | 24.03 | |
| Proportion of worst state “33333” | 0 | |
| **PedsQL GCS** |  |  |
| Total score | 67.17 (19.49) | 70.65 (14.13, 100) |
| Physical function score | 70.33 (22.89) | 75.00 (0, 100) |
| Psychosocial function score | 65.51 (19.55) | 66.67 (6.67, 100) |
| Emotional function score | 65.97 (21.44) | 65.00 (15.00, 100) |
| Social function score | 67.92 (24.71) | 70.00 (0, 100) |
| School function score | 62.46 (20.98) | 65.00 (0, 100) |
| Pain | 63.64 (27.28) | 50.00 (0, 100) |
| **Caregiver burden** |  |  |
| ZBI-22 total score | 34.31 (16.83) | 34.50 (0, 85) |

## Convergent validity

EQ-5D-Y utility had a strong correlation with the PedsQL GCS total score (rho = 0.72, *p*<0.001) and was moderately correlated with PedsQL GCS domains (rho range 0.59 to 0.68, *p*<0.001 for all) (Table 3). EQ-5D VAS was moderately correlated with PedsQL total score and domains (rho range, 0.47 to 0.60, *p*<0.001 for all).

For EQ-5D-Y dimensions and PedsQL GCS scales, which measure similar constructs, the EQ-5D-Y “mobility” and PedsQL GCS physical functioning were moderately correlated (rho = -0.45, *p*<0.001). Besides, “looking after him/herself”, “doing usual activities”, and “having pain or discomfort” in the EQ-5D-Y were moderately correlated (rho range, -0.42 to -0.60, *p*<0.001 for all) with the PedsQL GCS physical functioning. The EQ-5D-Y “feeling worried, sad or unhappy” and PedsQL GCS emotional functioning were moderately correlated (rho = -0.45, *p*<0.001). The EQ-5D-Y “doing usual activities” and PedsQL GCS school functioning were moderately correlated (rho = -0.42, *p*<0.001). “Having pain or discomfort” (rho = -0.56, *p*<0.001) and “feeling worried, sad or unhappy” (rho = -0.48, *p*<0.001) were moderately correlated with PedsQL GCS psychosocial health score. The psychosocial health score was calculated as the average of scores in emotional functioning, social functioning, and school functioning.

Table 3. Spearman's correlation coefficients between EQ-5D-Y and PedsQL GCS at domain level

|  | PedsQL GCS | | | | | |
| --- | --- | --- | --- | --- | --- | --- |
| EQ-5D-Y | Physical functioning | Emotional functioning | Social functioning | School functioning | Psychosocial health score | Total Score |
| Mobility | **-0.45^***^** | -0.25**^**^** | -0.25**^**^** | -0.19**^*^** | -0.26**^**^** | -0.36**^***^** |
| Looking after him/herself | **-0.42^***^** | -0.16**^*^** | -0.33**^***^** | -0.29**^***^** | -0.30**^***^** | -0.37**^***^** |
| Doing usual activities | **-0.58^***^** | -0.37**^***^** | **-0.48^***^** | **-0.42^***^** | **-0.49^***^** | -0.55**^***^** |
| Having pain or discomfort | **-0.60^***^** | -0.50**^***^** | -0.48**^***^** | -0.52**^***^** | **-0.56^***^** | -0.60**^***^** |
| Feeling worried, sad or unhappy | -0.28**^***^** | **-0.45^***^** | -0.40**^***^** | -0.42**^***^** | -**0.48^***^** | -0.43**^***^** |
| Utility | 0.68^***^ | 0.60^***^ | 0.60^***^ | 0.59^***^ | 0.68^***^ | **0.72^***^** |
| EQ-5D VAS | 0.59^***^ | 0.49^***^ | 0.47^***^ | 0.49^***^ | 0.55^***^ | **0.60^***^** |

Note: Values present in bold font indicate similar constructs.

* *p* < 0.05; ** *p* < 0.01; *** *p* < 0.001.

## Known-groups Validity

EQ-5D-Y utility scores of patients who had outpatient visits more than 5 times in 2021, who had lower PedsQL GCS total scores and scale scores or who had higher ZBI-22 total scores, were lower than their counterparts, with effect sizes of medium to large magnitude (Table 4) [58]. EQ-5D VAS scores of patients who had lower PedsQL GCS total scores and scale scores or who had higher ZBI-22 total scores, were lower than their counterparts, with effect sizes of medium to large magnitude.

Table 4. Known group analysis

| Known groups | EQ-5D-Y utility | EQ-5D VAS |
| --- | --- | --- |
| **Gender, mean (SD)** |  |  |
| Female (n=79) | 0.86 (0.16) | 75.03 (21.06) |
| Male (n=75) | 0.90 (0.10) | 75.76 (20.38) |
| Δ | -0.03 | -0.73 |
| ES (Cohen’s *d*) | -0.26 (-0.58, 0.06) | -0.04 (-0.35, 0.28) |
| Z | -0.98 | -0.14 |
| *p* value | 0.33 | 0.89 |
| **Annual outpatient visit times, mean (SD)^*^** |  |  |
| <5 (n=126) | 0.90 (0.11) | 75.77 (19.78) |
| >=5 (n=28) | 0.81 (0.20) | 73.64 (24.59) |
| Δ | 0.08 | 2.13 |
| ES (Cohen’s *d*) | 0.65 (0.24, 1.07) | 0.10 (-0.31, 0.51) |
| Z | 2.01 | 0.04 |
| *p* value | 0.04 | 0.84 |
| **Annual hospitalization times, mean (SD)^*^** |  |  |
| 0 (n=113) | 0.89 (0.13) | 76.08 (20.54) |
| >=1 (n=41) | 0.86 (0.14) | 73.46 (21.16) |
| Δ | 0.02 | 2.62 |
| ES (Cohen’s *d*) | 0.17 (-0.19, 0.53) | 0.13 (-0.23, 0.48) |
| Z | 0.91 | 0.78 |
| *p* value | 0.36 | 0.44 |
| **PedsQL Physical Function score, mean (SD)** |  |  |
| ≤75 (n=84) | 0.82 (0.15) | 66.31 (20.93) |
| >75 (n=70) | 0.95 (0.07) | 86.27 (14.12) |
| Δ | -0.13 | -19.96 |
| ES (Cohen’s *d*) | -1.11 (-1.45, -0.77) | -1.10 (-1.44, -0.76) |
| Z | -7.16 | -6.22 |
| *p* value | <0.0001 | <0.0001 |
| **PedsQL Emotional Function score, mean (SD)** |  |  |
| ≤65 (n=80) | 0.83 (0.13) | 67.43 (20.83) |
| >65 (n=74) | 0.93 (0.11) | 83.99 (16.77) |
| Δ | -0.10 | -16.56 |
| ES (Cohen’s *d*) | -0.85 (-1.18, -0.52) | -0.87 (-1.20, -0.54) |
| Z | -6.50 | -5.27 |
| *p* value | <0.0001 | <0.0001 |
| **PedsQL Social Function score, mean (SD)** |  |  |
| ≤70 (n=81) | 0.83 (0.13) | 68.91 (20.46) |
| >70 (n=73) | 0.94 (0.11) | 82.56 (18.52) |
| Δ | -0.11 | -13.65 |
| ES (Cohen’s *d*) | -0.90 (-1.23, -0.57) | -0.70 (-1.02, -0.37) |
| Z | -6.60 | -4.50 |
| *p* value | <0.0001 | <0.0001 |
| **PedsQL School Function score, mean (SD)** |  |  |
| ≤65 (n=85) | 0.83 (0.15) | 68.53 (21.16) |
| >65 (n=69) | 0.94 (0.07) | 83.83 (16.63) |
| Δ | -0.12 | -15.30 |
| ES (Cohen’s *d*) | -0.96 (-1.29, -0.62) | -0.79 (-1.12, -0.46) |
| Z | -6.18 | -4.87 |
| *p* value | <0.0001 | <0.0001 |
| **PedsQL pain, mean (SD)** |  |  |
| ≤50 (n=89) | 0.83 (0.15) | 69.15 (21.64) |
| >50 (n=65) | 0.95 (0.07) | 83.92 (15.77) |
| Δ | -0.12 | -14.78 |
| ES (Cohen’s *d*) | -0.96 (-1.30, -0.63) | -0.76 (-1.09, -0.43) |
| Z | -6.34 | -4.63 |
| *p* value | <0.0001 | <0.0001 |
| **ZBI-22, mean (SD)** |  |  |
| ≤34.5 (n=77) | 0.93 (0.09) | 82.70 (16.92) |
| >34.5 (n=77) | 0.83 (0.15) | 68.06 (21.57) |
| Δ | 0.10 | 14.64 |
| ES (Cohen’s *d*) | 0.83 (0.50, 1.15) | 0.76 (0.43, 1.08) |
| Z | 5.44 | 4.47 |
| *p* value | <0.0001 | <0.0001 |

^*^Information of annual outpatient visit times and hospitalization times in 2021 were collected.

Δ, the difference between the two groups.

ES, effect size, is expressed using Cohen’s d and its 95% confidence interval.

## Informativity and Discriminatory Power

The Shannon index and Shannon evenness index of the EQ-5D-Y dimensions were 0.60 and 0.38 for “mobility”, 0.79 and 0.50 for “looking after him/herself”, 0.98 and 0.62 for “doing usual activities”, 1.20 and 0.76 for “having pain or discomfort”, 1.23 and 0.78 for “feeling worried, sad or unhappy” (Table 5).

Table 5. Shannon index and Shannon evenness of EQ-5D-Y

|  | EQ-5D-Y | | | | |
| --- | --- | --- | --- | --- | --- |
|  | Mobility | Looking after him/herself | Doing usual activities | Having pain or discomfort | Feeling worried, sad or unhappy |
| Shannon index | 0.60 | 0.79 | 0.98 | 1.20 | 1.23 |
| Shannon evenness index | 0.38 | 0.50 | 0.62 | 0.76 | 0.78 |

# Discussion

Although measurement properties of HRQL measures in rare disease patients are increasingly the focus of researchers, the present study was the first study analyzing the measurement properties of EQ-5D-Y against PedsQL GCS among underage NF1 patients in China, to our knowledge. We observed the measurement properties of the EQ-5D-Y proxy version based on cross-sectional survey data which evaluated the HRQL among children with NF1, reported by their caregivers[59]. Reliable and widely recognized validation methods were employed. Drawing on data from a survey of 154 caregivers as proxies of children with NF1, EQ-5D-Y showed strong consistency with PedsQL GCS. The known-group hypotheses based on varying PedsQL GCS and ZBI scores were validated. The “feeling worried, sad, or unhappy” dimension of EQ-5D-Y displayed the highest Shannon index and Shannon evenness index. The findings indicate that the EQ-5D-Y is a reliable and efficient preference-based tool for evaluating HRQL in this population.

[67]According to a study validated pediatric preference-based HRQL instruments (EQ-5D-Y and CHU9D), validation of EQ-5D-Y was suggested to be conducted among the population with 10 diseases, including anxiety/depression, attention deficit hyperactivity disorder (ADHD), autism, and recurrent abdominal pain, three of which were related to pain, and some were closely associated with psychological health [60]. The common symptoms of these ten diseases align closely with the dimensions of EQ-5D-Y. Therefore, the author stated that these 10 diseases may be suitable for studies describing the measurement properties of EQ-5D-Y. Some underage NF1 patients have manifestations of pain, and clinical symptoms that affect their physical appearance may lead to psychological distress. These disease manifestations in patients with NF1 are aligned with the dimensions of EQ-5D-Y. Therefore, we inferred that underage NF1 patients may serve as an appropriate population for assessing the measurement properties of EQ-5D-Y. [66]

Based on the 154 study samples, the ceiling effect and the floor effect of EQ-5D-Y were 30.52% and 0%, respectively. The high ceiling effect observed in this study may be explained by the fact that many NF1 patients exhibit only mild skin symptoms. [68]In previous studies, ceiling effects of EQ-5D-Y proxy version were reported ranging from 45% to 68% in different populations, such as the general population, patients with renal and urinary diseases, or patients with metabolism and nutrition disorders [21, 33, 61-64].[21, 39, 69-71] The [21, 71]EuroQol Group developed EQ-5D-Y-5L, a revised version of EQ-5D-Y that extends the response levels from 3 to 5 levels in each dimension [65, 66].[26, 39, 48, 75] The EuroQol Group believes that EQ-5D-Y-5L would reduce the ceiling effects and enhance the sensitivity of EQ-5D-Y, which needs to be further assessed in NF1 population.[26, 48, 39]

As for convergent construct validity, results of the present study suggested that EQ-5D-Y proxy version has acceptable convergent construct validity in the population of underage NF1 patients. EQ-5D-Y utility and PedsQL GCS total score were highly related (Spearman’s rho = 0.72), exceeding previously reported correlation coefficients, which ranged from 0.20 (in mostly healthy children) to 0.66 (in acutely ill children) [19, 64, 67-70]. [19, 80, 81]This Spearman’s correlation coefficient observed in our study is on the upper bound of previous literature values. One possible reason is that, according to the calculation formula of Spearman’s correlation, the high ceiling effect of the EQ-5D-Y results in more frequent rank ties, which in turn lowers the Spearman correlation coefficient [71, 72]. Compared to our results, previous studies with a higher ceiling effect usually reported a lower Spearman’s correlation between EQ-5D-Y utility and PedsQL total scores [64, 69].

Results of known-groups validity indicated that EQ-5D-Y did have the ability to discriminate between underage patients defined by the subgroups which were set according to the presupposition that different HRQL levels were known to be observed. These hypotheses of known groups “annual outpatient visit times” and “annual hospitalization times” were not confirmed. We initially used these to reflect the severity of the disease indirectly because there was a lack of clinical information for the patients. The DNB classification (dermatological, neurological and bone manifestations) and imaging examination like magnetic resonance imaging (MRI) can tell the severity of NF1 in a more direct way[73]. It would be better if clinical information on NF1 patients, such as MRI and the number of neurofibromas, be collected to assess the severity of the disease in future studies on the performance of EQ-5D-Y.

In this study, we can only assess the measurement properties of EQ-5D-Y proxy version. Measuring HRQL is a complex process that requires consideration of various factors. Some HRQL measures showed good agreement and consistency between reports from proxies and self-reports, while others did not [29-31, 74]. For the same HRQL measure, proxy-reported versions may sometimes overestimate HRQL in certain populations and underestimate it in others [30, 75-77]. Caregivers, often acting as proxies, may have their assessments of HRQL influenced by factors such as their own gender and health status [76, 77]. For those patients over 8 years old, it is recommended that the self-reported version of EQ-5D-Y is a better choice [40]. How the older children perceive their health is important and can provide more direct information [78], which need to be investigated in future studies. [47]

# Limitations

There are some limitations in the present study. Only construct validity and discriminatory power could be examined since the study’s data were from a cross-sectional survey. Test-retest reliability needs to be assessed in future studies. Feasibility, which can be examined by the missing rate and the overall time taken to finish EQ-5D-Y, was not investigated in the present study since we use the one-on-one online design to conduct the survey and necessary questions of the EQ-5D-Y and PedsQL GCS were set as required questions, except for those questions that were claimed to be optional.

[89]For ethical reasons, only proxy versions of EQ-5D-Y and PedsQL GCS were used in this study. [47]We expected to investigate the performance of self-reported EQ-5D-Y and other HRQL instruments among NF1 patients in future studies.

# Conclusion

Overall, EQ-5D-Y is an acceptable choice to evaluate the HRQL of NF1 patients as it is a short preference-based HRQL measure, with significant advantages of time-saving, and results can be used to calculate quality-adjusted life years in economic evaluation. Further validation of HRQL measures among underage NF1 patients using longitudinal data and based on self-report data will provide more information. More evidence of validation and reliability of EQ-5D-Y among different populations is expected.

# Acknowledgments

We sincerely acknowledge the contribution of the volunteers from Neurofibromatosis Shenzhen Care Center during the data collection and support all the caregivers for underage NF1 patients who participated in the cross-sectional survey.

# Authors’ Contributions

XJ and WL contributed to the study design. Data collection was performed by WL, SC, YS, LZ, and LY. WL conducted the statistical analysis. The draft of the manuscript was written by WL under XJ’s supervision. XJ, HW, YS, and HW critically revised the manuscript. All authors read and approved the final manuscript.

# Funding

The work was based on the data collected from a cross-sectional survey sponsored by the China Alliance for Rare Diseases and the Beijing Society of Rare Disease Clinical Care and Accessibility. The work was also sponsored by Beijing Nova Program (No. Z211100002121060).

# Availability of data and materials

The datasets used in this study are not publicly available due to privacy concerns. However, interested individuals can request access to the data from the corresponding author, subject to approval by Beijing University of Chinese Medicine.

# List of abbreviations

ADHD, attention deficit hyperactivity disorder; EQ-5D, EuroQol five-dimensional; HTA, health technology assessment; HRQL, health-related quality of life; MRI, magnetic resonance imaging; NORD, National Organization for Rare Disorders; NSCC, Neurofibromatosis Shenzhen Care Center; NF1, neurofibromatosis type 1; PedsQL GCS, PedsQL 4.0 Generic Core Scales; QALYs, quality-adjusted life years; VAS, visual analog scale; ZBI-22, Zarit Burden Interview.

# Declarations

**Ethics approval and consent to participate**

The study was approved by the Ethics Committee of Beijing University of Chinese Medicine (2022BZYLL1005) and was carried out according to the Declaration of Helsinki. Electronically signed informed consent was obtained before the survey began.

**Consent for publication**

Not applicable.

# Competing interests

XJ is a member of EuroQol Group Association. Other authors declare that they have no conflict of interest.

# References

1. The Lancet Global H. The landscape for rare diseases in 2024. Lancet Glob Health. 2024;12(3):e341. doi:10.1016/S2214-109X(24)00056-1.

2. Melnikova I. Rare diseases and orphan drugs. Nature Reviews Drug Discovery. 2012;11(4):267-8. doi:10.1038/nrd3654.

3. Global Genes. RARE Disease Facts. 2023. <https://globalgenes.org/rare-disease-facts/>. Accessed September 25 2023.

4. Evans DG, Howard E, Giblin C, Clancy T, Spencer H, Huson SM et al. Birth incidence and prevalence of tumor-prone syndromes: estimates from a UK family genetic register service. Am J Med Genet A. 2010;152A(2):327-32. doi:10.1002/ajmg.a.33139.

5. Bergqvist C, Servy A, Valeyrie-Allanore L, Ferkal S, Combemale P, Wolkenstein P et al. Neurofibromatosis 1 French national guidelines based on an extensive literature review since 1966. Orphanet J Rare Dis. 2020;15(1):37. doi:10.1186/s13023-020-1310-3.

6. Xinhua. China releases second Catalog of rare diseases. 2023. <http://english.www.gov.cn/news/202309/21/content_WS650b7dc6c6d0868f4e8df9f1.html>.

7. EURORDIS-Rare Diseases Europe. List of the marketing authorisations. 2023. <https://www.eurordis.org/information-support/list-of-the-marketing-authorisations/>. Accessed September 25 2023.

8. Zhao Z, Pei Z, Hu A, Zhang Y, Chen J. Analysis of incentive policies and initiatives on orphan drug development in china: challenges, reforms and implications. Orphanet J Rare Dis. 2023;18(1):220. doi:10.1186/s13023-023-02684-8.

9. National Institute for health and Care Excellence (NICE). Single technology appraisal and highly specialised technologies evaluation: User guide for company evidence submission template. 202. <https://www.nice.org.uk/process/pmg24/chapter/cost-effectiveness>.

10. Shiroiwa T. Cost-effectiveness evaluation for pricing medicines and devices: A new value-based price adjustment system in Japan. Int J Technol Assess Health Care. 2020;36(3):270-6. doi:10.1017/S0266462320000264.

11. The Pharmaceutical Benefits Advisory Committee (PBAC). Guidelines for preparing submissions to the Pharmaceutical Benefits Advisory Committee (PBAC). 2016. <https://pbac.pbs.gov.au/information/about-the-guidelines.html>.

12. Swedish Dental and Pharmaceutical Benefits Agency (TLV). Swedish TLV report on its activities in health economic evaluations of medical technologies in 2020. 2020. <https://mtrconsult.com/news/swedish-tlv-report-its-activities-health-economic-evaluations-medical-technologies-2020>.

13. The Professional Society for Health Economics and Outcomes Research. Pharmacoeconomic guidelines around the world. 2023. <https://www.ispor.org/heor-resources/more-heor-resources/pharmacoeconomic-guidelines>. Accessed September 25 2023.

14. Kwon J, Freijser L, Huynh E, Howell M, Chen G, Khan K et al. Systematic Review of Conceptual, Age, Measurement and Valuation Considerations for Generic Multidimensional Childhood Patient-Reported Outcome Measures. Pharmacoeconomics. 2022;40(4):379-431. doi:10.1007/s40273-021-01128-0.

15. Bahrampour M, Jones R, Dalziel K, Devlin N, Mulhern B, Team Q. Comparing Generic Paediatric Health-Related Quality-of-Life Instruments: A Dimensionality Assessment Using Factor Analysis. Pharmacoeconomics. 2024;42(Suppl 1):81-94. doi:10.1007/s40273-024-01382-y.

16. The Professional Society for Health Economics and Outcomes Research. Pharmacoeconomic Guidelines: China Mainland. 2024. <https://www.ispor.org/heor-resources/more-heor-resources/pharmacoeconomic-guidelines/pe-guideline-detail/china-mainland>. Accessed December 28 2024.

17. Yang Z, Jiang J, Wang P, Jin X, Wu J, Fang Y et al. Estimating an EQ-5D-Y-3L value set for China. Pharmacoeconomics. 2022;40(Suppl 2):147-55. doi:10.1007/s40273-022-01216-9.

18. EuroQol Research Foundation. EQ-5D-Y-3L User Guide (Version 2.1). 2024. <https://euroqol.org/information-and-support/documentation/user-guides/>. Accessed December 28 2024.

19. Scott D, Ferguson GD, Jelsma J. The use of the EQ-5D-Y health related quality of life outcome measure in children in the Western Cape, South Africa: psychometric properties, feasibility and usefulness - a longitudinal, analytical study. Health Qual Life Outcomes. 2017;15(1):12. doi:10.1186/s12955-017-0590-3.

20. Kim SK, Jo MW, Kim SH. A cross sectional survey on health-related quality of life of elementary school students using the Korean version of the EQ-5D-Y. PeerJ. 2017;5:e3115. doi:10.7717/peerj.3115.

21. Pan CW, Zhong H, Li J, Suo C, Wang P. Measuring health-related quality of life in elementary and secondary school students using the Chinese version of the EQ-5D-Y in rural China. BMC Public Health. 2020;20(1):982. doi:10.1186/s12889-020-09116-3.

22. Sun Y, Zhou HJ, Shen A, Wu B, Wang W, Luo N et al. A cross-sectional study evaluating health-related quality of life of Chinese pediatric patients with hematological malignancies using EQ-5D-Y. Front Public Health. 2022;10:1050835. doi:10.3389/fpubh.2022.1050835.

23. Nematollahi S, Rampakakis E, Amara M, Hamdy RC, Rauch F, Hyer LC et al. Health-related quality of life in 205 children with arthrogryposis multiplex congenita. Qual Life Res. 2024. doi:10.1007/s11136-024-03808-8.

24. Sargenius HL, Ro TB, Hypher RE, Brandt AE, Andersson S, Finnanger TG et al. Health-related quality of life in children and adolescents with paediatric acquired brain injury: Secondary data analysis from a randomised controlled trial. Qual Life Res. 2024. doi:10.1007/s11136-024-03838-2.

25. Hu J, Zhu L, Bao H, Liu Y, Xing H, Kang Q et al. Utility estimations of different health states of patients with type I, II, and III spinal muscular atrophy in China: a mixed approach study with patient and proxy-reported data. Front Public Health. 2022;10:1054931. doi:10.3389/fpubh.2022.1054931.

26. Xu RH, Zhu L, Sun R, Tan RL, Luo N, Zou S et al. Investigating the psychometric properties of the EQ-5D-Y-3L, EQ-5D-Y-5L, CHU-9D, and PedsQL in children and adolescents with osteogenesis imperfecta. Eur J Pediatr. 2022;181(12):4049-58. doi:10.1007/s00431-022-04626-1.

27. Lin J, Wong CKH, Cheung JPY, Cheung PWH, Luo N. Psychometric performance of proxy-reported EQ-5D youth version 5-level (EQ-5D-Y-5L) in comparison with three-level (EQ-5D-Y-3L) in children and adolescents with scoliosis. Eur J Health Econ. 2022;23(8):1383-95. doi:10.1007/s10198-022-01435-z.

28. Wong CKH, Cheung PWH, Luo N, Lin J, Cheung JPY. Responsiveness of EQ-5D Youth version 5-level (EQ-5D-5L-Y) and 3-level (EQ-5D-3L-Y) in patients with idiopathic scoliosis. Spine (Phila Pa 1976). 2019;44(21):1507-14. doi:10.1097/brs.0000000000003116.

29. Smith SC, Hendriks AAJ, Cano SJ, Black N. Proxy reporting of health-related quality of life for people with dementia: a psychometric solution. Health Qual Life Outcomes. 2020;18(1):148. doi:10.1186/s12955-020-01396-y.

30. Williams LS, Bakas T, Brizendine E, Plue L, Tu W, Hendrie H et al. How valid are family proxy assessments of stroke patients' health-related quality of life? Stroke. 2006;37(8):2081-5. doi:10.1161/01.STR.0000230583.10311.9f.

31. Pickard AS, Johnson JA, Feeny DH, Shuaib A, Carriere KC, Nasser AM. Agreement between patient and proxy assessments of health-related quality of life after stroke using the EQ-5D and Health Utilities Index. Stroke. 2004;35(2):607-12. doi:10.1161/01.STR.0000110984.91157.BD.

32. Zhang H, Wang L, Quan M, Huang J, Wu P, Lu Q et al. Health-related quality of life in children with chronic immune thrombocytopenia in China. Health Qual Life Outcomes. 2016;14:45. doi:10.1186/s12955-016-0445-3.

33. Fitriana TS, Purba FD, Stolk E, Busschbach JJV. EQ-5D-Y-3L and EQ-5D-Y-5L proxy report: psychometric performance and agreement with self-report. Health Qual Life Outcomes. 2022;20(1):88. doi:10.1186/s12955-022-01996-w.

34. Shiroiwa T, Fukuda T, Shimozuma K. Psychometric properties of the Japanese version of the EQ-5D-Y by self-report and proxy-report: reliability and construct validity. Qual Life Res. 2019;28(11):3093-105. doi:10.1007/s11136-019-02238-1.

35. Gusi N, Perez-Sousa MA, Gozalo-Delgado M, Olivares PR. Validity and reliability of the spanish EQ-5D-Y proxy version. An Pediatr (Barc). 2014;81(4):212-9. doi:10.1016/j.anpedi.2013.11.028.

36. Ji Y, Chen S, Li K, Xiao N, Yang X, Zheng S et al. Measuring health-related quality of life in children with cancer living in mainland China: feasibility, reliability and validity of the Chinese mandarin version of PedsQL 4.0 Generic Core Scales and 3.0 Cancer Module. Health Qual Life Outcomes. 2011;9:103. doi:10.1186/1477-7525-9-103.

37. Sood S, Lim R, Collins L, Trajanovska M, Hutson JM, Teague WJ et al. The long-term quality of life outcomes in adolescents with Hirschsprung disease. J Pediatr Surg. 2018;53(12):2430-4. doi:10.1016/j.jpedsurg.2018.08.036.

38. Sluys KP, Lannge M, Iselius L, Eriksson LE. Six years beyond pediatric trauma: child and parental ratings of children's health-related quality of life in relation to parental mental health. Qual Life Res. 2015;24(11):2689-99. doi:10.1007/s11136-015-1002-y.

39. Sundaram SS, Alonso EM, Haber B, Magee JC, Fredericks E, Kamath B et al. Health related quality of life in patients with biliary atresia surviving with their native liver. J Pediatr. 2013;163(4):1052-7 e2. doi:10.1016/j.jpeds.2013.04.037.

40. EuroQol Research Foundation. EQ-5D-Y User Guide. How to apply, score, and present results from the EQ-5D-Y (Version 2.0). 2020. <https://euroqol.org/publications/user-guides/>. Accessed September 25 2023.

41. Pei W, Yue S, Zhi-Hao Y, Ruo-Yu Z, Bin W, Nan L. Testing measurement properties of two EQ-5D youth versions and KIDSCREEN-10 in China. Eur J Health Econ. 2021;22(7):1083-93. doi:10.1007/s10198-021-01307-y.

42. Mayoral K, Rajmil L, Murillo M, Garin O, Pont A, Alonso J et al. Measurement properties of the online EuroQol-5D-youth instrument in children and adolescents with type 1 diabetes mellitus: questionnaire study. J Med Internet Res. 2019;21(11):e14947. doi:10.2196/14947.

43. Zhou W, Shen A, Yang Z, Wang P, Wu B, Herdman M et al. Patient-caregiver agreement and test-retest reliability of the EQ-5D-Y-3L and EQ-5D-Y-5L in paediatric patients with haematological malignancies. Eur J Health Econ. 2021;22(7):1103-13. doi:10.1007/s10198-021-01309-w.

44. Varni JW, Seid M, Rode CA. The PedsQL: measurement model for the pediatric quality of life inventory. Med Care. 1999;37(2):126-39. doi:10.1097/00005650-199902000-00003.

45. Nutakki K, Varni JW, Swigonski NL. PedsQL Neurofibromatosis Type 1 Module for children, adolescents and young adults: feasibility, reliability, and validity. J Neurooncol. 2018;137(2):337-47. doi:10.1007/s11060-017-2723-2.

46. Wolters PL, Vranceanu AM, Thompson HL, Martin S, Merker VL, Baldwin A et al. Current recommendations for patient-reported outcome measures assessing domains of quality of life in neurofibromatosis clinical trials. Neurology. 2021;97(7 Suppl 1):S50-S63. doi:10.1212/WNL.0000000000012421.

47. Dhaenens BAE, Rietman A, Husson O, Oostenbrink R. Health-related quality of life of children with neurofibromatosis type 1: analysis of proxy-rated PedsQL and CHQ questionnaires. Eur J Paediatr Neurol. 2023;45:36-46. doi:10.1016/j.ejpn.2023.05.010.

48. Kaartina S, Chin YS, Fara Wahida R, Woon FC, Hiew CC, Zalilah MS et al. Adolescent self-report and parent proxy-report of health-related quality of life: an analysis of validity and reliability of PedsQL 4.0 among a sample of Malaysian adolescents and their parents. Health Qual Life Outcomes. 2015;13:44. doi:10.1186/s12955-015-0234-4.

49. Varni JW, Seid M, Kurtin PS. PedsQL 4.0: reliability and validity of the Pediatric Quality of Life Inventory version 4.0 generic core scales in healthy and patient populations. Med Care. 2001;39(8):800-12. doi:10.1097/00005650-200108000-00006.

50. Seng BK, Luo N, Ng WY, Lim J, Chionh HL, Goh J et al. Validity and reliability of the Zarit Burden Interview in assessing caregiving burden. Ann Acad Med Singap. 2010;39(10):758-63.

51. Lu L, Wang L, Yang X, Feng Q. Zarit Caregiver Burden Interview: development, reliability and validity of the Chinese version. Psychiatry Clin Neurosci. 2009;63(6):730-4. doi:10.1111/j.1440-1819.2009.02019.x.

52. Ko KT, Yip PK, Liu SI, Huang CR. Chinese version of the Zarit caregiver Burden Interview: a validation study. Am J Geriatr Psychiatry. 2008;16(6):513-8. doi:10.1097/JGP.0b013e318167ae5b.

53. Yang X, Yoo HK, Amin S, Cheng WY, Sundaresan S, Zhang L et al. Burden among caregivers of pediatric patients with neurofibromatosis type 1 (NF1) and plexiform neurofibroma (PN) in the United States: a cross-sectional study. Neurol Ther. 2022;11(3):1221-33. doi:10.1007/s40120-022-00365-5.

54. Adib-Hajbaghery M, Ahmadi B. Caregiver burden and its associated factors in caregivers of children and adolescents with chronic conditions. Int J Community Based Nurs Midwifery. 2019;7(4):258-69. doi:10.30476/IJCBNM.2019.73893.0.

55. Javalkar K, Rak E, Phillips A, Haberman C, Ferris M, Van Tilburg M. Predictors of caregiver burden among mothers of children with chronic conditions. Children (Basel). 2017;4(5). doi:10.3390/children4050039.

56. Scalone L, Tomasetto C, Matteucci MC, Selleri P, Cavrini G. Assessing quality of life in children and adolescents: development and validation of the Italian version of the EQ-5D-Y. Epidemiology Biostatistics & Public Health. 2011;8(4):331-41.

57. Akoglu H. User's guide to correlation coefficients. Turk J Emerg Med. 2018;18(3):91-3. doi:10.1016/j.tjem.2018.08.001.

58. Lakens D. Calculating and reporting effect sizes to facilitate cumulative science: a practical primer for t-tests and ANOVAs. Front Psychol. 2013;4:863. doi:10.3389/fpsyg.2013.00863.

59. Liang W, Cao S, Suo Y, Zhang L, Yang L, Wang P et al. Long-term distress throughout one's life: health-related quality of life, economic and caregiver burden of patients with neurofibromatosis type 1 in China. Front Public Health. 2024;12:1398803. doi:10.3389/fpubh.2024.1398803.

60. Xiong X, Dalziel K, Huang L, Mulhern B, Carvalho N. How do common conditions impact health-related quality of life for children? Providing guidance for validating pediatric preference-based measures. Health Qual Life Outcomes. 2023;21(1):8. doi:10.1186/s12955-023-02091-4.

61. Ravens-Sieberer U, Wille N, Badia X, Bonsel G, Burström K, Cavrini G et al. Feasibility, reliability, and validity of the EQ-5D-Y: results from a multinational study. Qual Life Res. 2010;19(6):887-97. doi:10.1007/s11136-010-9649-x.

62. Mulhern B, Meadows K. The construct validity and responsiveness of the EQ-5D, SF-6D and Diabetes Health Profile-18 in type 2 diabetes. Health Qual Life Outcomes. 2014;12:42. doi:10.1186/1477-7525-12-42.

63. Oluboyede Y, Tubeuf S, McCabe C. Measuring health outcomes of adolescents: report from a pilot study. Eur J Health Econ. 2013;14(1):11-9. doi:10.1007/s10198-011-0340-0.

64. Konomura K, Numakura C, Nakamura-Utsunomiya A, Hoshino E, Tajima G, Kobayashi H et al. Health-related quality of life and caregiver burden of pediatric patients with inborn errors of metabolism in Japan using EQ-5D-Y, PedsQL, and J-ZBI. Qual Life Res. 2024;33(12):3323-33. doi:10.1007/s11136-024-03775-0.

65. Kreimeier S, Astrom M, Burstrom K, Egmar AC, Gusi N, Herdman M et al. EQ-5D-Y-5L: developing a revised EQ-5D-Y with increased response categories. Qual Life Res. 2019;28(7):1951-61. doi:10.1007/s11136-019-02115-x.

66. EuroQol Research Foundation. EQ-5D-Y-5L User Guide (Version 1.0). 2024. <https://euroqol.org/information-and-support/documentation/user-guides/>.

67. Xu RH, Zhao Z, Mao Z, Wang S, Xiong H, Dong D. Measurement properties of the EQ-5D-Y-3L, PedsQL 4.0, and PROMIS-25 Profile v2.0 in pediatric patients with spinal muscular atrophy. Health Qual Life Outcomes. 2024;22(1):50. doi:10.1186/s12955-024-02264-9.

68. López-Bastida J, López-Siguero JP, Oliva-Moreno J, Vázquez LA, Aranda-Reneo I, Reviriego J et al. Health-related quality of life in type 1 diabetes mellitus pediatric patients and their caregivers in Spain: an observational cross-sectional study. Curr Med Res Opin. 2019;35(9):1589-95. doi:10.1080/03007995.2019.1605158.

69. Fitriana TS, Purba FD, Stolk E, Busschbach JJV. Indonesia youth population norms for EQ-5D-Y-3 L, EQ-5D-Y-5 L and the PedsQL generic core scale: lower health related quality of life relates to high economic status and stress. BMC Public Health. 2023;23(1):1124. doi:10.1186/s12889-023-16003-0.

70. He S, Chen L, Wang P, Xi X. Psychometric Comparison of EQ-5D-Y, CHU-9D, and PedsQL 4.0 in Chinese Children and Adolescents With Functional Dyspepsia: A Multicenter Study. Value Health. 2023;26(12):1754-62. doi:10.1016/j.jval.2023.08.007.

71. Wissler C. The Spearman Correlation Formula. Science. 1905;22(558):309-11. doi:10.1126/science.22.558.309.

72. Sijtsma K, Emons WHM. Nonparametric Statistical Methods. In: Peterson P, Baker E, McGaw B, editors. International Encyclopedia of Education (Third Edition). Oxford: Elsevier; 2010. p. 347-53.

73. Ehara Y, Yamamoto O, Kosaki K, Yoshida Y. Clinical severity in Japanese patients with neurofibromatosis 1 based on DNB classification. J Dermatol. 2017;44(11):1262-7. doi:10.1111/1346-8138.13902.

74. Chen P, Hudson MM, Li M, Huang IC. Correction: Health utilities in pediatric cancer patients and survivors: a systematic review and meta‑analysis for clinical implementation. Qual Life Res. 2023;32(4):1221. doi:10.1007/s11136-022-03273-1.

75. Russell KM, Hudson M, Long A, Phipps S. Assessment of health-related quality of life in children with cancer: consistency and agreement between parent and child reports. Cancer. 2006;106(10):2267-74. doi:10.1002/cncr.21871.

76. Rosser SA, Lloyd M, Hu A, Loke P, Tang MLK. Associations between gender and health-related quality of life in people with IgE-mediated food allergy and their caregivers: A systematic review. Clin Exp Allergy. 2024;54(2):93-108. doi:10.1111/cea.14450.

77. Hung SY, Pickard AS, Witt WP, Lambert BL. Pain and depression in caregivers affected their perception of pain in stroke patients. J Clin Epidemiol. 2007;60(9):963-70. doi:10.1016/j.jclinepi.2006.12.010.

78. Matza LS, Patrick DL, Riley AW, Alexander JJ, Rajmil L, Pleil AM et al. Pediatric patient-reported outcome instruments for research to support medical product labeling: report of the ISPOR PRO good research practices for the assessment of children and adolescents task force. Value Health. 2013;16(4):461-79. doi:10.1016/j.jval.2013.04.004.
